# Structural basis for the activation and inhibition of Sirtuin 6 by quercetin and its derivatives

**DOI:** 10.1038/s41598-019-55654-1

**Published:** 2019-12-16

**Authors:** Weijie You, Wei Zheng, Sandra Weiss, Katrin F. Chua, Clemens Steegborn

**Affiliations:** 1 Department of Biochemistry, University of Bayreuth, 95445 Bayreuth, Germany; 2 Department of Medicine, Stanford University School of Medicine, Stanford, CA 94305 USA; 3 Geriatric Research, Education, and Clinical Center, Veterans Affairs Palo Alto Health Care System, Palo Alto, CA 94304 USA

**Keywords:** Enzymes, Structural biology, Biochemistry, Chemical biology, Natural products, Small molecules

## Abstract

Mammalian Sirtuin 6 (Sirt6) is an NAD^+^-dependent protein deacylase regulating metabolism and chromatin homeostasis. Sirt6 activation protects against metabolic and aging-related diseases, and Sirt6 inhibition is considered a cancer therapy. Available Sirt6 modulators show insufficient potency and specificity, and even partially contradictory Sirt6 effects were reported for the plant flavone quercetin. To understand Sirt6 modulation by quercetin-based compounds, we analysed their binding and activity effects on Sirt6 and other Sirtuin isoforms and solved crystal structures of compound complexes with Sirt6 and Sirt2. We find that quercetin activates Sirt6 via the isoform-specific binding site for pyrrolo[1,2-a]quinoxalines. Its inhibitory effect on other isoforms is based on an alternative binding site at the active site entrance. Based on these insights, we identified isoquercetin as a ligand that can discriminate both sites and thus activates Sirt6 with increased specificity. Furthermore, we find that quercetin derivatives that inhibit rather than activate Sirt6 exploit the same general Sirt6 binding site as the activators, identifying it as a versatile allosteric site for Sirt6 modulation. Our results thus provide a structural basis for Sirtuin effects of quercetin-related compounds and helpful insights for Sirt6-targeted drug development.

## Introduction

Sirtuins are NAD^+^-dependent protein lysine deacylases that sense the cellular nutrient status, regulate energy metabolism and stress responses, and have been implicated in aging processes and aging-related diseases^1^. Of the seven mammalian Sirtuin isoforms, Sirt1–7, three are localized in the nucleus (Sirt1,6,7). Sirt1 is the most studied isoform, functions as deacetylase of histones and transcription factors, and it is modulated by a variety of small molecule inhibitors and activators and used as a therapeutic target^2^. Sirt6 associates with chromatin and modulates the functions of histones, transcription factors, and stress response proteins to regulate gene expression, telomere maintenance, and DNA repair^3^. Sirt6 promotes longevity in male mice, and it suppresses aging phenotypes and induces apoptosis in cancer cells^4–6^. Small molecule Sirt6 modulators are thus sought for functional studies and therapy of aging-related diseases, such as cancer and type 2 diabetes^7^.

Sirtuins feature differing substrate acyl selectivities^8–11^. *In vitro*, Sirt6 shows low deacetylase activity on peptides and free histones and more efficient turnover of long chain acylations, such as myristoylations^8^. However, Sirt6 efficiently deacetylates nucleosomal histones *in vitro*, and nucleosomes as well as proteins involved in glucose metabolism and DNA repair in cells^7,12^. It features the conserved Sirtuin catalytic core of ~275 amino acids, with a Rossman-fold and a Zn^2+^-binding domain^10^. Sirt6’s particular small Zn^2+^-binding domain and cofactor-binding loop render its substrate acyl-binding channel rather large and hydrophobic, facilitating accommodation of long-chain acyls. The Sirtuin catalytic core is flanked by isoform-specific N- and C- terminal extensions, which in Sirt6 contribute to its chromatin association^13^.

**Figure 1:**
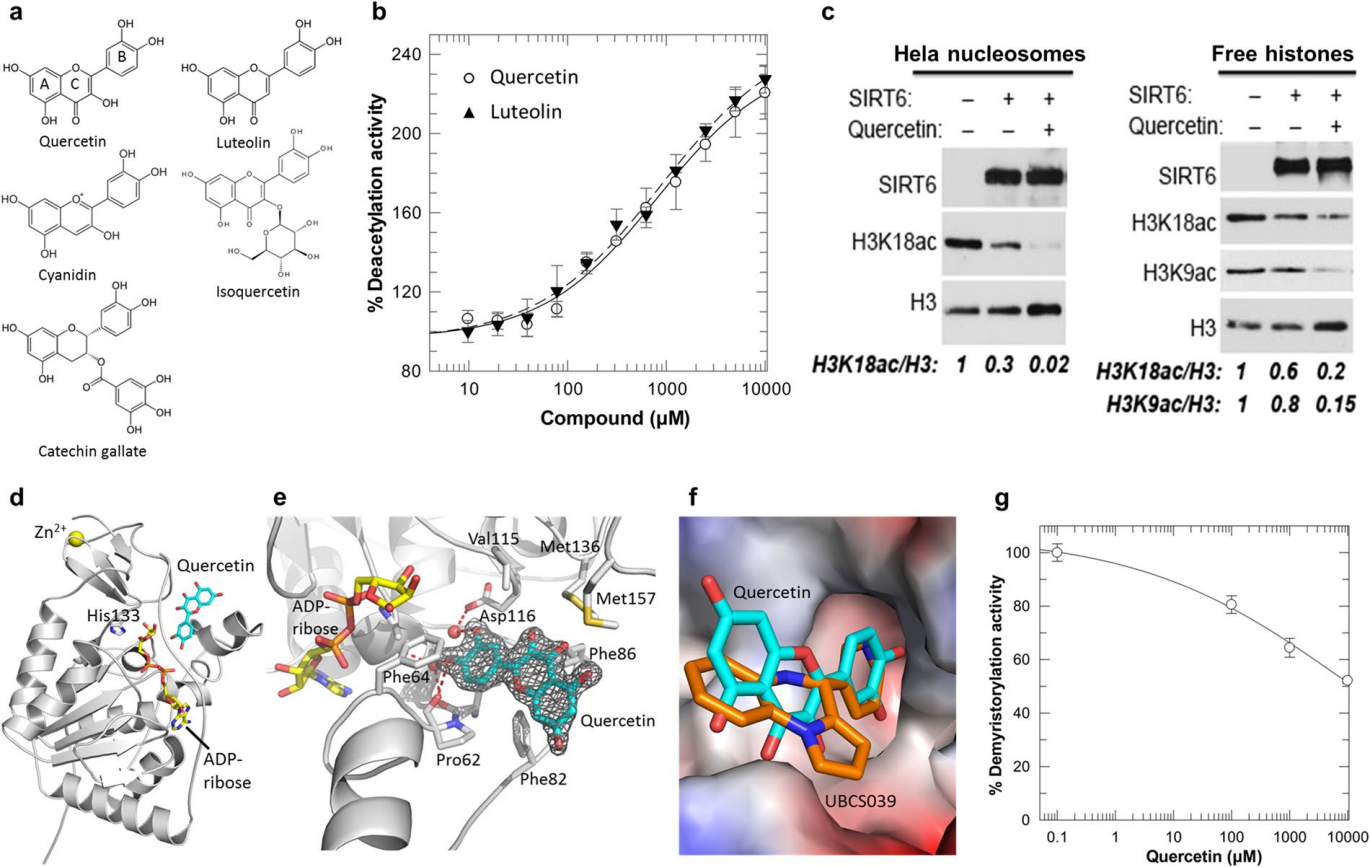
Activation of Sirt6-dependent deacetylation and crystal structure of a Sirt6/quercetin complex. **(a)** Chemical structures of quercetin and its derivatives. **(b)** Dose-dependent effects of quercetin and luteolin on Sirt6 deacetylation activity. (n = 3; error bars: s.d.) **(c)** Western blots showing deacetylation activity of SIRT6 on purified Hela cell nucleosomes (left) or free histones (right), and activation by quercetin (5 mM) compared to DMSO vehicle control. Blots were cropped for detection of the indicated entities and are representative of 3 independent experiments. Relative acetylation on the indicated sites was determined by normalization to total H3 and control samples. **(d)** Overall structure of the complex between human Sirt6 (cartoon), ADP-ribose (yellow sticks), and quercetin (cyan sticks); His133 is shown as sticks to indicate the active site. **(e)** Interaction of quercetin with Sirt6. Interacting residues are labeled, and polar interactions are indicated by dashed red lines. 2F_o_-F_c_ electron density for the ligand is contoured at 1σ. **(f)** Protein surface of the Sirt6/quercetin complex colored according to the electrostatic potential. The ligand is shown as cyan sticks and overlayed with UBCS039 (orange sticks). **(g)** Titration with quercetin inhibits Sirt6-dependent demyristoylation. (n = 3; error bars: s.d.).

Only few Sirt6 modulators are available. Initially described Sirtuin-activating compounds (STACs) bind to a Sirt1-specific STAC binding domain (SBD)^2^, suggesting that activation might only be possible for Sirt1. However, free fatty acids and fatty acid ethanolamides at high micromolar concentrations were found to stimulate Sirt6 deacetylation activity^14,15^. They inhibit Sirt6-dependent hydrolysis of long chain acylations, implicating the acyl binding channel in this effect^14^. More recently, we identified synthetic pyrrolo[1,2-a]quinoxaline derivatives as more potent Sirt6 deacetylase activators, albeit with limited efficacy and solubility^16,17^. Crystal structures of Sirt6 complexes with these activators identified a Sirt6-specific modulator binding site in the acyl binding channel^17^. The anti-inflammatory and anti-diabetic plant flavonoid quercetin (**1**; Fig. 1a)^18,19^ appears to be another Sirt6 activator. Quercetin was reported to activate Sirt1 and to inhibit Sirt6 in the “Fluor-de-Lys” (FdL) assay employing a fluorogenic substrate^20,21^. Quercetin has further been described as Sirt6 inhibitor at low concentrations^15,22^ and Sirt6 activator at high concentrations^15^ based on mass spectrometry (MS) assays, whereas a recent MS study yielded only activating effects for quercetin and a more potent derivative^23^. It further indicated the quercetin derivatives catechin gallate (**2**; Fig 1a) and gallocatechin gallate (**3**) as potent small molecule Sirt6 inhibitors (IC_50_ 2.5 and 5.4 µM, respectively), the first ones besides the non-specific trichostatin A^24,25^. Quercetin and its derivatives affect several cellular targets^19^, but Sirtuin binding site and regulation mechanisms would nevertheless improve our understanding of Sirt6 modulation and be helpful for the development of potent and soluble Sirt6 modulators. So far, pharmacophore models^22,26^ and docking calculations^23^ failed to rationalize the limited and partially contradictory experimental data on Sirt6 modulation.

Here, we analyse modulation of Sirt6 and other isoforms by quercetin and its derivatives in activity and binding assays and through crystal structure analyses of modulator complexes of Sirt6 and Sirt2. We find that quercetin-based compounds can activate Sirt6-dependent deacetylation through binding to the Sirt6-specific acyl binding channel. We further show that quercetin inhibits other Sirtuin isoforms through an alternative binding site at the active site entrance, and that isoquercetin (**4**; Fig. 1a) can distinguish these binding sites and thus activates Sirt6 selectively. Interestingly, we find that the inhibitory quercetin derivatives exploit the same Sirt6 binding site as the activators, but with a rotated chromen-4-one orientation within the acyl binding channel. Our results hence reveal sites and mechanisms for Sirtuin modulation by quercetin derivatives and provide helpful insights for Sirt6 modulator development.

## Results

### Quercetin and its derivative luteolin activate Sirt6-dependent deacetylation

To clarify the effects of quercetin and its derivatives on Sirt6 deacetylation activity, we tested them in several assays. In the FdL assay, quercetin appeared to inhibit Sirt6 but control reactions showed that it quenches FdL fluorescence, preventing reliable measurements (Supplementary Fig. 1a). In a coupled enzymatic assay with acetylated H3K9 peptide (H3K9ac), which represents a physiological Sirt6 deacetylation site, quercetin also appeared to inhibit Sirt6 but control reactions revealed suppressive quercetin effects on the downstream enzymes of the assay (Supplementary Fig. 1b). To overcome the artifacts in these widely used assays, we tested the compounds in a robust MS-based assay^27^ with the H3K9Ac substrate. Quercetin and luteolin (**5**; Fig. 1a) yielded a dose-dependent increase in Sirt6 activity, with a > 2-fold maximum stimulation and EC_50_ values of ~1.2 mM (Fig. 1b). We did not observe any evidence for the reported Sirt6 inhibition by lower quercetin concentrations^15,22^ and conclude that quercetin and luteolin act as low potency activators of Sirt6 peptide deacetylation activity.

Acetyl-peptides are poor Sirt6 substrates and may not fully mimic physiological deacetylations in cells^12,28^. We therefore examined quercetin effects on the deacetylation activity of Sirt6 on purified full-length histones and nucleosomes (Fig. 1c). Quercetin substantially activated Sirt6 in deacetylating nucleosomal H3K18ac (H3K9ac levels were below the detection limit). Moreover, quercetin also augmented the H3K18ac and H3K9ac deacetylase activities of Sirt6 on free full-length histones (Fig. 1c), which are otherwise poor substrates for Sirt6. Quercetin thus activates Sirt6-dependent deacetylation of its physiological histone substrates.

### A Sirt6/quercetin crystal structure reveals binding site and interaction details

To identify Sirt6’s quercetin binding site and interaction details, we determined a crystal structure of a Sirt6/ADP-ribose/quercetin complex at 1.84 Å resolution (Table 1; Fig. 1d,e). Well-defined electron density for the quercetin ligand revealed the distal end of Sirt6’s extended acyl binding channel as binding site (Fig. 1d–f). The catechol moiety of quercetin (ring B) is buried in a protein pocket and its 4’-hydroxyl group forms a hydrogen bond to the Pro62 backbone oxygen, and the 4′- and 3'-hydroxyls form water-mediated interactions with the backbone of Ala53 and Ile61 and the side chain of Asp116 (Fig. 1e,f; Supplementary Fig. 1c). Asp116 normally forms a hydrogen bond to the NAM moiety of NAD^+^ bound in the so-called “C-site”, illustrating that catechol pocket and C-site partly overlap. The quercetin chromen-4-one system contributes to complex formation through hydrophobic contacts with funnel surface patches formed by Phe64/82/86 and Val70/115, and Met136/157 (Fig. 1e,f). Comparison of the Sirt6/quercetin complex with a Sirt6 complex with the pyrrolo[1,2-a]quinoxaline-based activator UBCS039 shows that they share most of their binding sites (Fig. 1f). The catechol group of quercetin overlays well with the UBCS039 pyridine moiety and reproduces its key interaction to Pro62^17^. The chromen-4-one substitutes for the hydrophobic pyrrolo[1,2-a]quinoxalines of UBCS039-related compounds, which can vary between UBCS039 derivatives due to the non-directed nature of its interactions and the wide and hydrophobic architecture of the Sirt6 funnel^17^. It thus appears that the buried catechol group of quercetin functions as an anchor in Sirt6 binding, while the chromen-4-one provides smaller and non-specific binding contributions and might be particularly amenable for modifications to increase compound affinity.

**Table 1:** Diffraction data and refinement statistics.

	Sirt6 and quercetin	Sirt6 and isoquercetin	Sirt6 and CG	Sirt6 and cyanidin	Sirt2 and quercetin
**Data collection**					
Space group	P6_3_	P6_3_	P6_3_	P6_3_	P2_1_2_1_2_1_
Cell dimensions					
*a* = *b*, *c* (Å)	91.4, 143.9	91.4, 144.2	91.8, 144.2	91.4, 143.8	78.2, 114.5
Resolution (Å)^a^	47.98–1.84 (1.95–1.84)	45.71–1.90 (2.01–1.90)	48.12–2.01 (2.14–2.01)	47.94–2.10 (2.22–2.10)	46.22–2.23 (2.37–2.23)
*R*_merge_^a^	0.12 (1.76)	0.10 (1.63)	0.16 (1.90)	0.16 (1.84)	0.28 (1.90)
CC_1/2_^a^	0.999 (0.479)	0.999 (0.378)	0.996 (0.710)	0.998 (0.634)	0.976 (0.282)
*I* / σ*I* ^a^	11.6 (1.1)	13.2 (1.2)	9.7 (1.3)	11.0 (1.3)	4.4 (0.7)
Completeness (%)^a^	99.7 (98.5)	99.9 (99.3)	98.9 (96.2)	99.9 (99.5)	99.5 (97.1)
Redundancy ^a^	6.9 (6.9)	6.9 (6.6)	10.1 (8.5)	11.3 (11.3)	7.4 (7.4)
**Refinement**					
Resolution (Å) ^a^	45.68–1.84 (1.89–1.84)	43.57–1.90 (1.95–1.90)	45.96–2.01 (2.07–2.01)	45.69–2.10 (2.15–2.10)	46.22–2.23 (2.29–2.23)
No. reflections	56832	51587	42989	37769	32823
*R*_work_ / *R*_free_ (%)	16.8/20.3	15.3/18.7	16.3/19.3	16.3/20.0	21.1/23.0
Twin law^b^	None	−k, −h, −l	−k, −h, −l	−k, −h, −l	−k, −h, −l
Twin fractions^b^	n.a.	0.72/0.28	0.56/0.44	0.74/0.26	0.78/0.22
No. atoms					
Protein	4382	4381	4350	4341	4735
Compound	44	66	64	42	22
ADP ribose	72	72	72	72	72
Water	346	224	166	117	28
*B*-factors					
Protein	38.9	46.7	41.9	54.0	53.9
Compound	59.3	77.9	51.2	80.1	67.3
ADP ribose	28.7	36.1	30.2	41.8	34.8
Water	43.9	46.2	38.0	48.5	46.4
R.m.s. deviations					
Bond lengths (Å)	0.013	0.014	0.015	0.012	0.014
Bond angles (°)	1.84	2.11	2.26	1.95	2.2

^a^Highest-resolution shell is shown in parentheses.

^b^Twinning was detected through L-tests with CCP4 POINTLESS and twin fractions were determined during amplitude-based twin refinement with Refmac.

Since quercetin occupies the distal end of the extended Sirt6 acyl channel it is compatible with binding of acetylated substrates but would overlap with longer substrate acylations such as myristoylations (Supplementary Fig. 1d). Testing quercetin on Sirt6-dependent demyristoylation indeed revealed a concentration-dependent inhibition (Fig. 1g), consistent with results from continuous Sirt6 assays with a quencher-containing long chain acyl substrate^29^. Thus, quercetin stimulates the deacetylation activity of Sirt6 and inhibits Sirt6-dependent demyristoylations, as observed for UBCS039 and consistent with their binding to the remote end of the Sirt6 acyl channel.

### Sirt6 complex structures with activating and inhibitory quercetin derivatives reveal interaction differences and suggest modulation mechanisms

**Figure 2:**
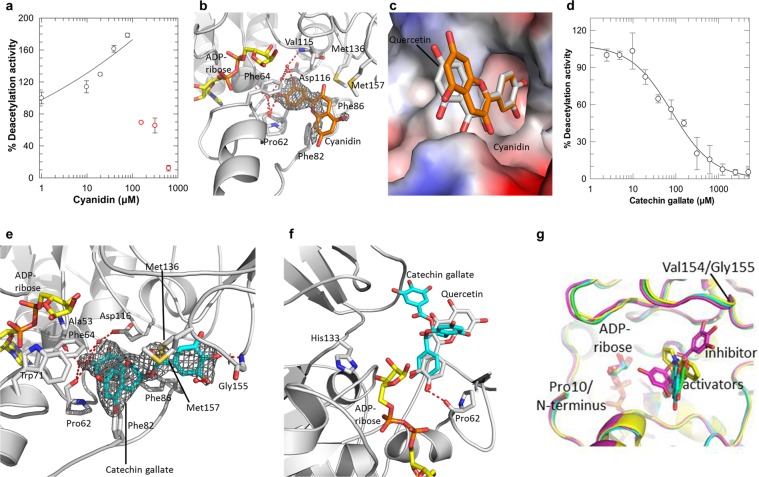
Effects of quercetin derivatives on Sirt6 and crystal structures of Sirt6/quercetin derivative complexes. **(a)** Dose-dependent effects of cyanidin on Sirt6 deacetylation activity. Samples corresponding to red data points showed precipitation and were excluded for the fitting curve shown. (n = 3; error bars: s.d.) **(b)** Complex of Sirt6 with cyanidin. Interacting residues are labeled, and polar interactions are indicated by dashed lines. 2Fo-Fc electron density for the ligand is contoured at 1σ. **(c)** Protein surface of the Sirt6/cyanidin complex colored according to the electrostatic potential. The ligand is shown as orange sticks and overlayed with quercetin (gray sticks). **(d)** Dose-dependent effects of CG on Sirt6 deacetylation activity. (n = 3; error bars: s.d.) **(e)** Complex of Sirt6 with CG. Interacting residues are labeled, and polar interactions are indicated by dashed lines. CG is covered with 2Fo-Fc density contoured at 1σ. (**f**) Overlay of Sirt6 complexes with CG (cyan) and quercetin (gray), respectively. ADP-ribose (yellow) and His133 at the active site are shown as sticks. (**g**) Overlay of Sirt6 complexes with the activators quercetin (green), cyanidin (cyan), UBCS039 (yellow) and the inhibitor CG (magenta).

Cyanidin (**6**; Fig. 1a) is an quercetin derivative that was reported to activate Sirt6 with slightly increased potency and efficacy (EC_50_ 460 ± 20 µM; 55-fold stimulation)^23^. In our activity assays, cyanidin caused precipitations at higher concentrations, preventing reliable measurements. It showed already significant Sirt6 activation at lower concentrations (20–80 μM; Fig. 2a), however, indeed indicating a higher potency than for quercetin. To obtain insights in activation mechanism and compound features relevant for the improved potency, we solved a crystal structure of a Sirt6/ADP-ribose/cyanidin complex (Table 1; Fig. 2b; Supplementary Fig. 2a). The activator is well-defined by electron density for its B-ring catechol, but features weaker density for the A and C ring system, which could have functional relevance but might also be caused by the lower solubility (and resulting occupancy) of cyanidin. The A/C rings nevertheless can be positioned unequivocally, in the same sites as for quercetin (Fig. 2c), and the compounds thus show the same binding mode.

Catechin gallate (CG) is the most potent quercetin derivative reported to inhibit rather than activate Sirt6-dependent deacetylation (IC_50_ 2.5 ± 0.1 μM)^23^. Testing CG in our MS assay confirmed the surprising inhibitory effect of this closely quercetin-related compound, albeit with slightly lower potency (IC_50_ 80 ± 15 μM; Fig. 2d). To rationalize the inhibitory effect, we determined a crystal structure of a Sirt6/CG complex at 2.0 Å resolution (Table 1; Fig. 2e). The bound inhibitor CG is well-defined by electron density and occupies the same binding region as the activator quercetin, with identical positions for their catechol moieties but a rotated chromen-4-one in GC to accommodate its additional, bulky trihydroxy benzoyl group (Fig. 2e,f; Supplementary Fig. 2b). The chromen-4-one now interacts with Trp71 of the acyl channel exit, and the trihydroxy benzoyl group forms hydrophobic interactions with the other side of the exit funnel and a polar contact to the Gly155 backbone. The inhibitor does not overlap with the binding pocket for acetyl substrate but the catechol group assumes a position partly overlapping with the NAM moiety of the NAD^+^ cosubstrate (Supplementary Fig. 2c). Testing CG inhibition at different NAD^+^ concentrations did not result in significant changes of residual activity (Supplementary Fig. 2d), however, indicating that the inhibition is not based on NAD^+^ competition. The fact that closely related compounds with identical catechol binding mode act as activators indeed suggests that the modulatory binding occurs after the NAM release step of the catalytic cycle.

The keto group of quercetin at C-ring position 4 is missing in the more potent activator cyanidin and the potent inhibitor CG, which removes an unfavourable interaction - with Met157 in case of cyanidin - and might be a key for their increased potency. Comparing Sirt6 complexes of activating and inhibiting compounds further suggests modulation mechanisms and differences relevant for their differing effects (Fig. 2g). The inhibitor CG differs from activators in a slight rotation of the catechol moiety, and more drastically in a tilted position of the chroman and the presence of its additional bulky substituent, orienting them toward N-terminus and Val154, respectively. The Val154/Gly155 peptide bond flips to provide space, but this and other differences in protein conformation are subtle. The bulky group pointing toward the N-terminus is CG’s dihydrochroman, and its strong tilt compared to quercetin/cyanidin is due to the saturated C-ring in CG. Interestingly, activating quercetin derivatives appear to feature an unsaturated, planar C-ring, while inhibiting family members tend to have saturated C-rings (our data and^15^) and thus likely bind in the tilted, CG-like orientation. The Sirt6-activating compound UBCS039^17^ orients its pyrrolo[1,2-a]quinoxaline similar to the chromene of the activating quercetin derivatives (Fig. 2g), which supports the relevance of this moiety position for activation. Both inhibiting and activating quercetin derivatives (and pyrrolo[1,2-a]quinoxalines) overlap with the C-site that accommodates the NAM moiety of NAD^+^ until NAM is released in the first catalytic step^10^. This binding mode implies for activators that they bind during or after NAM release. Such a mechanism was characterized for the potent Sirtuin inhibitor Ex-527 and also suggested for trichostatin A^24,30^, and it is indicated for the inhibitory quercetin derivatives by the lack of NAD^+^ competition (see above). It thus appears that the quercetin-based activators and inhibitors share this mechanistic feature, consistent with their similarity in chemical structure and binding mode. Different effects on the stability or conformational details of the acyl channel of Sirt6/product complexes, possibly due to the inhibitor’s chroman group pointing toward the N-terminus (Fig. 2g), might cause their differing effects on Sirt6 deacetylase activity, but details remain to be established.

### Quercetin inhibits other Sirtuin isoforms by exploiting an alternative binding site

**Figure 3:**
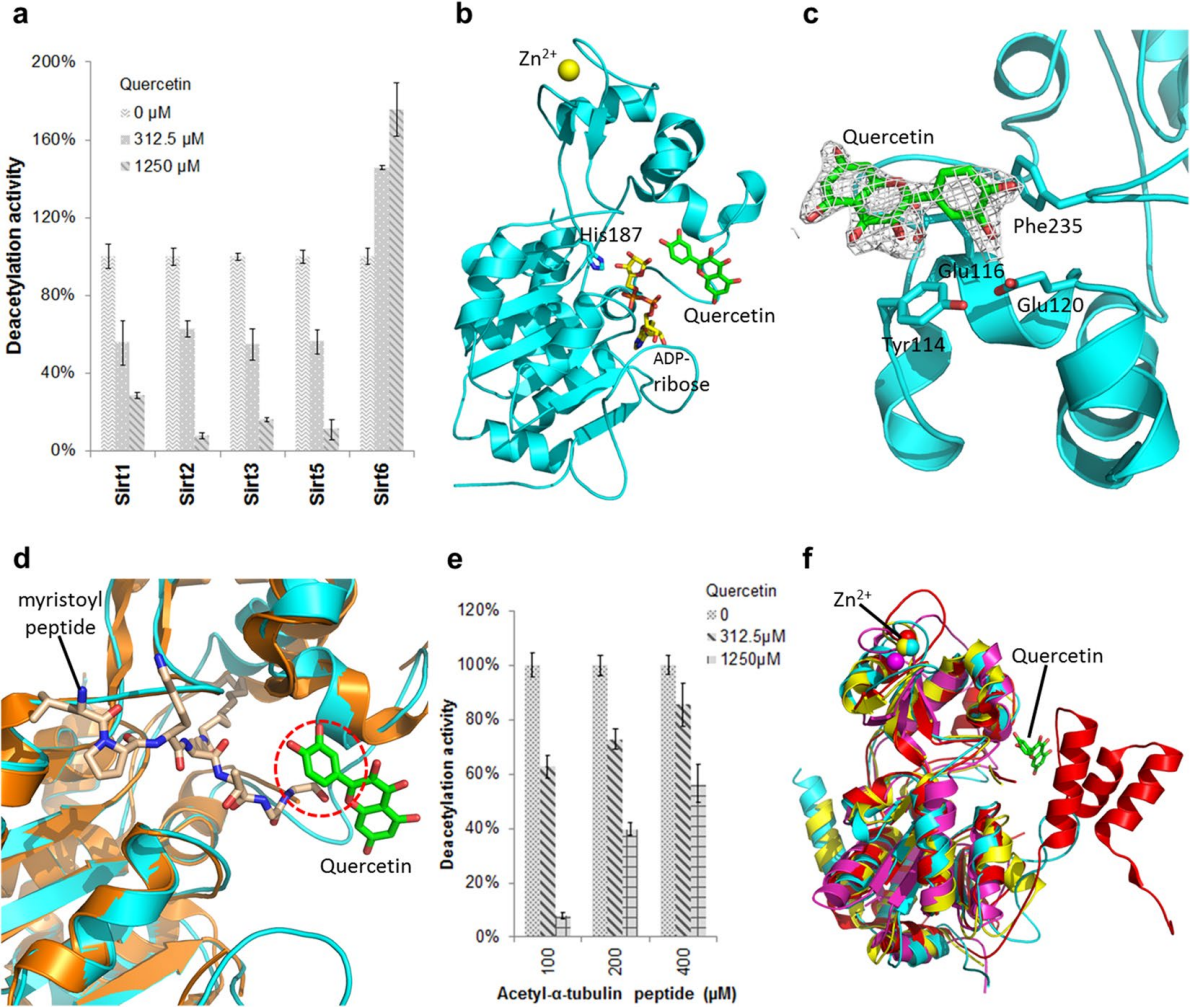
Effect of quercetin on Sirtuin isoforms and structure of Sirt2 in complex with quercetin. (**a)** Quercetin effects on the deacylation activities of Sirtuin isoforms. (n = 3; error bars: s.d.) **(b**) Overall structure of human Sirt2 (cartoon presentation), ADP-ribose (yellow sticks), and quercetin (green sticks); His187 at the active site is represented in sticks. **(c)** Close view of the Sirt2/quercetin complex, interacting residues are labeled and quercetin (green sticks) is covered with 2Fo-Fc density contoured at 1σ. **(d)** Overlay of the Sirt2/quercetin complex (cyan cartoon, green ligand) with a Sirt2/myristoyl-peptide complex (gold cartoon, wheat peptide; PDB code 4Y6O)^50^. Dotted circle: steric clash between substrate and quercetin. **(e)** Effects of quercetin on Sirt2 deacetylase activity at various substrate concentrations. (n = 3; error bars: s.d.) **(f)** Overlay of the Sirt2/quercetin complex (cyan) with Sirt1 (red; PDB code 5BTR)^51^, Sirt3 (yellow; PDB code 4HD8)^37^ and Sirt5 (magenta; PDB code 4HDA)^37^.

Due to the isoform-specific features of the Sirt6 acyl channel, in particular its wide and hydrophobic architecture^10,31^, its ligands should be Sirt6 specific. An overlay of the Sirt6/quercetin complex with Sirt1,2,3, and 5 indeed shows that the binding site is blocked in these other isoforms by cofactor loop and a helix bundle (Supplementary Fig. 3a). However, effects of quercetin and related polyphenols were also reported for Sirt1^20^. We thus tested the quercetin isoform specificity in activity assays with human Sirt1, Sirt2, Sirt3, Sirt6 (all with acetylated substrate), and Sirt5 (with succinylated substrate). Quercetin caused a concentration-dependent inhibition of all Sirtuin isoforms except for Sirt6, which was instead activated as described above (Fig. 3a). Due to the lack of a Sirt6-like binding site in the other isoforms, quercetin has to exploit an alternative binding site to cause this opposite, inhibitory effect.

To identify the alternative quercetin binding site and modulation mechanism, we determined a crystal structure of a Sirt2/quercetin complex at 2.2 Å resolution (Table 1; Fig. 3b,c). Well-defined electron density revealed that quercetin binds at the Sirt2 active site entrance, through π-π stacking with Tyr-114 and Phe-235 and anion–π interactions with Glu-116 and Glu-120 (Table 1; Fig. 3c; Supplementary Fig. 3b). Interestingly, quercetin is located between two symmetry-related Sirt2 monomers and interacts with the same surface region of the second monomer, albeit with rotated orientation and through fewer contacts (Supplementary Fig. 3c), suggesting the interaction with the first monomer to be the one relevant in solution. An overlay with a Sirt2/substrate peptide complex reveals that quercetin partly occupies the pocket accommodating substrate moieties C-terminal from the acetyl-Lys and thus sterically prevents substrate binding (Fig. 3d). Such a peptide competitive mechanism is supported by Sirt2 inhibition tests that show that increasing substrate concentrations lead to weaker inhibitory effects of a fixed inhibitor concentration (Fig. 3e).

Comparing the Sirt2/quercetin complex to other Sirtuin isoforms reveals that its binding site is also accessible in Sirt1, 3, and 5, whereas it is occupied in Sirt6 by the N-terminus (Fig. 3f; Supplementary Fig. 3d). Since the quercetin binding site of Sirt6 is blocked in these other isoforms, our structures define two mutually exclusive modulator binding sites for Sirt6 and for Sirt1,2,3,5, respectively.

### Isoquercetin is an activating ligand for the quercetin site with improved specificity

**Figure 4:**
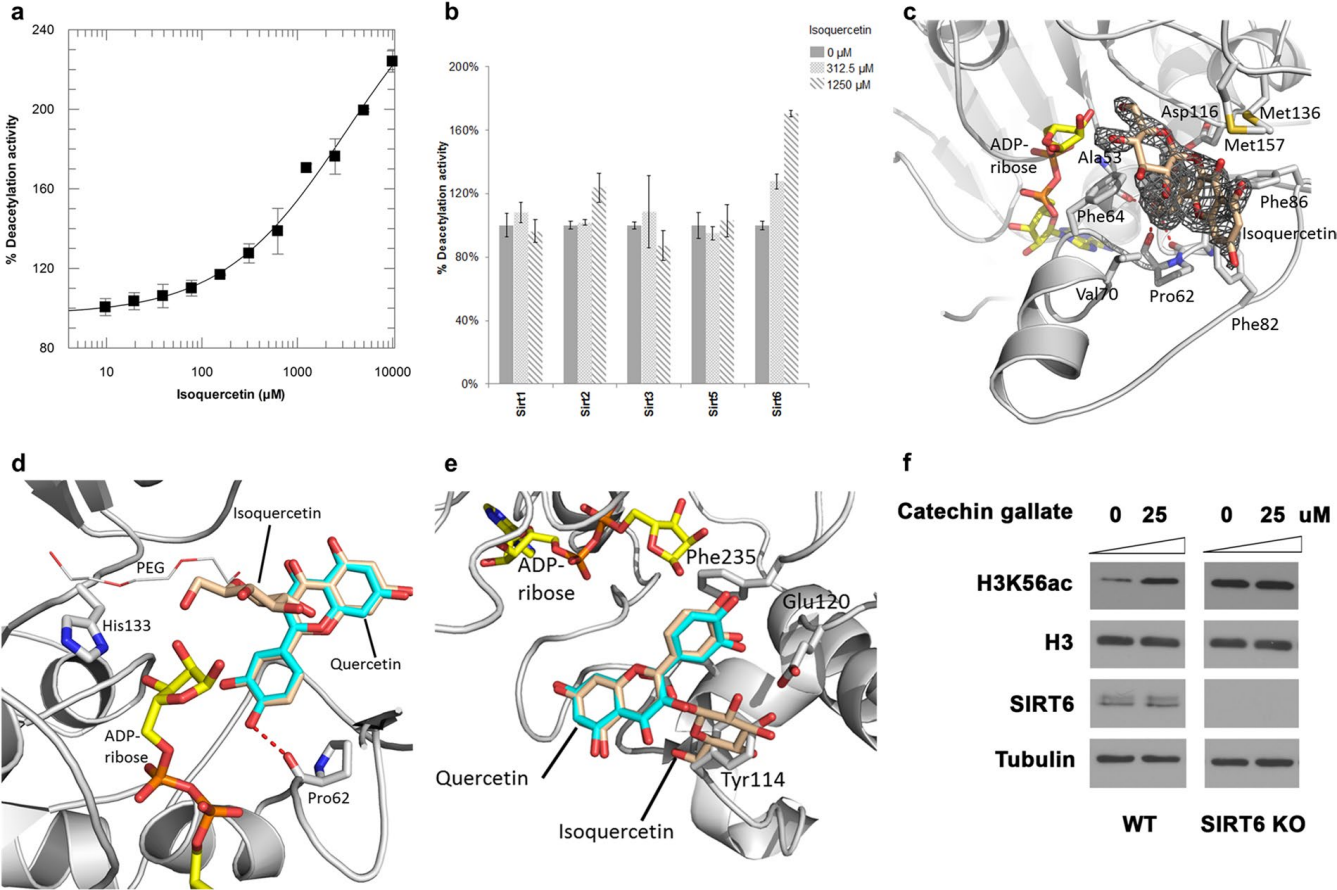
Isoquercetin activates Sirt6 with improved isoform specificity. **(a)** Dose-dependent effect of isoquercetin on Sirt6 deacetylation activity. (n = 3; error bars: s.d.) **(b)** Effect of isoquercetin on the deacylation activities of Sirtuin isoforms. (n = 3; error bars: s.d.) **(c)** Close view of the Sirt6/isoquercetin complex. Interacting residues are labeled and isoquercetin (wheat) is covered with 2Fo-Fc density contoured at 1σ. **(d)** Overlay of the Sirt6 complexes with quercetin (cyan) and isoquercetin (wheat), respectively (protein of isoquercetin complex omitted for clarity). **(e)** Quercetin-based overlay of the Sirt2 quercetin complex (grey cartoon, cyan sticks) and the beforehand superposed Sirt6 complexes with quercetin (not shown) and isoquercetin (wheat), respectively (Sirt6 protein omitted for clarity). **(f)** Histone deacetylation activity in cells. After treatment with CG at indicated doses for 48 hours, acetylation levels of H3K56 were analysed by Western blots. Blots were cropped for detection of the indicated entities.

Since the quercetin sites of Sirt6 and Sirt1,2,3,5 are mutually exclusive, suitable derivatives should allow to exploit specific features of either site and thus show improved selectivity. We thus tested the isoform selectivity of the quercetin derivative isoquercetin (Fig. 1a), which features a bulky sugar moiety that might be accommodated in the acyl channel of Sirt6 but not in the alternative site. Isoquercetin indeed retained the Sirt6 stimulating activity, albeit with even lower potency, and it showed no significant effects on Sirt1–3-dependent deacetylation and Sirt5-dependent desuccinylation (Fig. 4a,b). To confirm the molecular basis of the improved Sirt6 selectivity of isoquercetin, we determined a crystal structure of a Sirt6/ADP-ribose/isoquercetin complex. Initial soaking experiments failed due to a PEG molecule bound to the acyl channel, but substituting PEG with ethylene glycol enabled us to solve the Sirt6/isoquercetin complex structure at 1.8 Å resolution (Table 1; Fig. 4c). The quercetin moiety of isoquercetin occupies the activator site identical to the parent compound (Fig. 4d; Supplementary Fig. 3e), and its additional sugar moiety is thereby placed in the acyl channel. The density for the isoquercetin sugar is weaker than for its quercetin moiety, likely due to flexibility, and it indeed forms no significant positive interactions (Fig. 4c; Supplementary Fig. 3e). Overlaying isoquercetin with the ligand of our Sirt2/quercetin structure illustrates that the sugar moity would clash with Tyr-114, rationalizing the improved selectivity for Sirt6 (Fig. 4e). The sugar at this compound position thus acts as a negative selector, i.e. does not disturb Sirt6 binding significantly but hinders accommodation in the alternative binding site of the other isoforms, indicating that other moieties at this position should result in further improvements in selectivity and – with more appropriate moieties for beneficial interactions – in better potency.

## Discussion

Sirt6 is considered as therapeutic target for aging-related diseases^1,3^, but the few available Sirt6 modulators require improvements concerning potency, selectivity, and bioavailability^2,24^. Partly contradictory effects on Sirt6 activity were previously reported for the plant flavone quercetin^15,21,22,26^, and we find that the compound interferes with two popular Sirtuin activity assays, which is a common problem for optical assays^32^. Our robust MS assay confirms quercetin as a low potency Sirt6 activator. Previously reported inhibitory effects at lower quercetin concentrations could not be observed and likely are assay artifacts caused by the optical effects of the compound and/or the use of Sirt6 tagged with GST, which is known to bind quercetin tightly^33^. We could confirm, however, that some quercetin derivatives act as Sirt6 inhibitors rather than activators, and the slight differences in potency might again be attributed to the previous use of a GST tag^23^. The more potent quercetin-based Sirt6 modulators^23^, together with our results, suggest that potency and specificity of this scaffold can be significantly increased and improved compounds might eventually even become suitable for pharmacological applications. They further indicate that Sirt6 modulation might contribute to physiological effects of such more potent compound family members. Indeed, treating U2OS cancer cells with the potent inhibitor CG led to significant histone H3 hyperacetylation, an established marker for Sirt6 inhibition, and this effect was abolished in SIRT6-deficient cells (Fig. 4f)^34^. The Sirt6 activating quercetin derivatives showed low potency, and quercetin itself affects more potently a variety of other cellular targets, such as kinases^19^. Sirt6 might still contribute to the beneficial health effects of quercetin, but potentially indirectly, through its activation by increased NAD^+^ levels from quercetin inhibition of CD38^35^. Nonetheless, we found that treatment of U2OS cells with 25 to 100 μM quercetin induced a dose-dependent decrease of H3 acetylation that was not observed in SIRT6-deficient cells, demonstrating clear Sirt6-dependent cellular effects of higher quercetin concentrations (Supplementary Fig. 3f). Direct Sirt6 activation thus might contribute to physiological effects observed at higher quercetin concentrations. Importantly, the more potent quercetin derivatives, such as CG, indicate that Sirt6-activating and –inhibiting natural quercetin derivatives with even further increased potency might exist and that Sirt6 modulation might contribute to effects of compound family members and thus of flavone-containing foods or extracts. Due to the wide Sirt6 binding side and few specific interactions, even other natural compound families with a catechol group or a related moiety could exploit this binding mode. They might be the explanation why the deacetylase activity of Sirt6 is weak *in vitro* yet significant *in vivo*^7^. Furthermore, quercetin inhibits other Sirtuin isoforms with low potency and more potent derivatives appear to exist also for these isoforms^36^ and might contribute to physiological effects of the compound family.

For exploiting insights from studying quercetin-based Sirtuin modulators for drug development, Sirtuin/compound interaction details are required. A pharmacophore model based on binding competition suggested four relevant hydrogen bonds^26^. Docking models indicated binding in active site and acyl channel, with inhibitors disturbing substrate binding and a more external binding of activators^23^. Both modelings were based on limited and partially incorrect data (see above). Our crystal structures in fact identify a deviating Sirt6 binding mode and provide interaction details and modulation mechanisms. Activators and inhibitors bind almost identical, with the catechol moiety acting as an anchor. It binds at the bottom of the Sirt6-specific acyl channel, similar to the pyridine moiety of activating pyrrolo[1,2-a]quinoxalines^17^. The resulting overlap with the C-site indicates that the modulatory binding takes place only after NAM release^32^. The chroman/chromene moieties contribute mostly non-directional hydrophobic contacts and thus can assume different orientations within the Sirt6 acyl funnel. They are structurally distinct from the corresponding pyrrolo[1,2-a]quinoxalines groups and more polar, revealing that these parts of the two compound families should be amenable to significant modifications that might improve affinity and/or solubility. Within the quercetin family, variations would mainly be restricted by the observation that saturation state and substituents of the chroman/chromene decide its orientation and thereby seem to determine whether a ligand activates or inhibits. It will be interesting to see which other scaffolds, besides pyrrolo[1,2-a]quinoxalines, can imitate either binding mode and possibly facilitate the development of highly potent and soluble allosteric Sirt6 activators and inhibitors, respectively. Furthermore, C-ring modifications in quercetin derivatives, respective corresponding positions in pyrrolo[1,2-a]quinoxalines or potential other ligand classes, appear in particular suitable for increasing Sirt6 selectivity due to their incompatibility with the newly identified quercetin binding site in Sirt1,2,3,5.

Our structures reveal alternative binding sites in Sirt6 respective Sirt1,2,3,5, and thereby explain quercetin’s Sirt6-specific activation effect and its weak inhibition of other isoforms. The comparison of quercetin in both sites reveals C-ring modifications, as in isoquercetin, as a feature that can increase Sirt6 selectivity and possibly also affinity, and the Sirt6/CG complex reveals how quercetin derivatives can also exploit this site for potent Sirt6 inhibition. In principle, our structures also provide information for improving the inhibitory binding to other Sirtuin isoforms, but strong and specific binding might be difficult to achieve due to their related, rather flat and exposed sites. In summary, our results hence provide structural and mechanistic insights in Sirtuin modulation by quercetin-based compounds and will support for further development of Sirt6 activators and inhibitors.

## Materials and Methods

### Chemicals

All chemicals were purchased from Sigma if not stated differently. All synthetic peptides were from GL Biochem (myristoyl-TNF: EALPK-(myristoyl-K)-TGG); acetyl-H3K9: MARTKQTAR-(acetyl-K)-STGGKAPRKQL; acetyl-p53: RHK-(acetyl-K)-LMFK; acetyl-a-tubulin: MPSD-(acetyl-K)-TIG; acetyl-ACS2: TRSG-(acetyl-K)-VMR; succinyl-CPS1: RGVL-(succinyl-K)-EYGV).

### Protein production and purification

A comparison of the protein constructs we used is shown in Supplementary Fig. 4. The recombinant N-terminal his-tagged human Sirt6 proteins were produced as previously described^16^. Briefly, Sirt6(1-355) in pQE80L.1 was fermented in *E. coli* M15[pREP4]; Sirt6(13–308) in pET151-D-TOPO was expressed in *E. coli* Rosetta2 (DE3) pLysS. Human Sirt2(55–356) was expressed from a pET-SUMO vector in E. coli BL21 (DE3) codon + . The proteins were purified by affinity chromatography with Talon resin (Clontech), followed by tag cleavage with Tobacco Etch Virus (TEV) protease. Tag and protease were removed through a second Talon affinity chromatography, and the proteins were further purified using cation exchange and gel filtration chromatography. Purified protein was concentrated to 10 mg/ml for Sirt6 and 36.5 mg/ml for Sirt2, flash frozen in liquid nitrogen, and stored at −80 °C. Full length human Sirt1, human Sirt3 residues 118–399, and human Sirt5 residues 34–302 were prepared as described before^16,37^.

### Peptide deacylation assays

For coupled enzymatic peptide deacylation assays, reactions were run in a total volume of 100 µl containing 50 mM Na-phosphate pH 7.50, 5% DMSO, 0.6 mM DTT, 0.1% (v/v) Tween 20, 200 µM acetylated histone H3K9 peptide, 500 µM NAD^+^ and 10 µM Sirt6. The reactions were monitored in an Epoch 2 plate reader (BioTek) at 340 nm wavelength. Control reactions to check for compound effects on downstream enzymes contained no Sirt6 and were spiked with 40 µM nicotinamide.

For FdL assays, reactions were run in a total volume of 50 µl containing 50 mM Tris-HCl pH 7.50, 100 mM NaCl, 5% DMSO, 100 µM acetylated FdL1-peptide, 500 µM NAD^+^ and 10 µM Sirt6. After incubation at 37 °C for 1 h, reactions were stopped by adding 2 mM NAM and 10 mg/ml trypsin, incubated 20 min, and measured in a FluoDia T70 (Photon Technology) at wavelength 460 nm. Control reactions for fluorescence quenching effects of the compounds were run by adding compound at different concentrations after the deacetylation and development steps.

For MS deacetylation assays, reactions contained 50 mM Na-phosphate pH 7.5, 200 µM H3K9ac peptide, 2.5 mM NAD^+^, 5% DMSO, the indicated amount of compound and 20 µM Sirt6(1–355). Demyristoylation assays were done with 50 µM myristoyl-TNFα peptide. Reference reactions contained 5% DMSO and no compound, and control reactions were run without Sirt6. After incubation for 2 h at 37 °C, reactions were stopped by adding equal volumes of 0.5% (v/v) trifluoroacetic acid and diluted 10-fold with 0.1% formic acid. Samples were filtered in 10 kDa MWCO concentrators and analyzed on an LTQ-XL mass spectrometer (Thermo Scientific) coupled to an HPLC-system with a self-packed ReproSil-Pur C18-AQ column. Demyristoylation samples were analyzed on a TripleTOF 5600 + System (ABI Sciex) coupled to an HPLC-system with a Jupiter 5 u C4 300 A column (Phenomenex) without prior filtration. Peptide quantification was done with Skyline^38^. For the Sirt1, 2, 3, and 5 deacylation assays, the reaction mixtures contained 100 µM acetyl-p53 (Sirt1), 100 µM acetyl-α-tubulin (Sirt2), 100 µM acetyl-ACS2 (Sirt3), or 100 µM succinyl-CPS1 (Sirt5), respectively. Sirt3 samples contained in addition 0.05 mg/ml nicotinamidase. All reactions further contained 500 µM NAD^+^ and indicated amounts of compounds in 50 mM Na-phosphate buffer with 5% DMSO and were incubated for 5 min at 37 °C. MS analyses of the peptides were done as described for Sirt6.

### *In vitro* histone and nucleosome deacetylation assays

2 μg GST or GST-Sirt6 protein was pre-incubated with 5 mM quercetin or DMSO vehicle at room temperature for 10 minutes. *In vitro* deacetylation reactions were then performed by adding 5 μg calf thymus histones or 2 μg HeLa mononucleosomes (Epicypher) in NAD^+^ deacetylation buffer (20 mM Tris pH 8, 150 mM NaCl, 1 mM NAD^+^, 1.5 mM DTT), incubated at 30 °C for 3 hours in a total volume of 20 μl. Histone deacetylation was assessed by western blot with H3K9ac (ab4441) or H3K18ac (ab1191) specific antibodies. Signals were quantified with Image Studio Lite software (LI-COR Biosciences), and relative acetylation determined by normalization to total H3 and control samples.

### Cellular deacetylation assays

Human U2OS cells (ATCC) were cultured in EMEM with 10% FBS and Pen/Strep. SIRT6 KO U2OS cells were generated by CRISPR/Cas9 gRNA targeting SIRT6 as described previously^39^. Cells were treated with CG or quercetin at indicated doses for 48 hours, and whole-cell lysates were collected to determine histone acetylation levels in cells. Western blot analysis of histone acetylation levels was performed with anti-H3K56ac (Epitomics 2134–1) antibody.

### Crystallization and Structure determination

Sirt6/ADP-ribose crystals were grown from 1.6 M (NH_4_)_2_SO_4_, 10% PEG 400, and Bis-Tris buffer pH 5.7 with 10 mg/ml Sirt6(13–308) and 10 mM ADP-ribose by the hanging drop vapor diffusion method at 20 °C^17^. The Sirt6/ADPr/quercetin and Sirt6/ADP-ribose/cyanidin complexes were obtained by soaking Sirt6/ADP-ribose crystals with 40 mM compound for one week, and the Sirt6/ADP-ribose/CG complex by soaking with 1 mM CG overnight. The structure in complex with isoquercetin was produced by transferring crystals in a new drop containing 1.6 M (NH_4_)_2_SO_4_, 10% ethylene glycol, and Bis-Tris buffer pH 5.7 with 40 mM isoquercetin and incubation for one week. A solution of reservoir supplemented with 20% ethylene glycol, 10 mM compound and 2 mM ADP-ribose was used as cryoprotectant.

Sirt2/ADP-ribose crystals were grown in hanging drops at 20 °C with 14% PEG 10.000 and 0.1 M ammonium acetate pH 5.8 as reservoir solution^40^. The protein solution contained 13 mg/ml Sirt2 (55–356) and 20 mM ADP-ribose. The Sirt2/ADP-ribose crystals were soaked with 40 mM quercetin for 3 days. Crystals were then transferred to a drop of reservoir supplemented with 20% glycol, 10 mM quercetin and 2 mM ADP-ribose before flash freezing in liquid nitrogen.

Diffraction data were collected at 100 K at BL14.1 operated by Helmholtz-Zentrum Berlin (HZB) at the BESSY II electron storage ring (Berlin-Adlershof, Germany)^41^. Diffraction data were processed with the X-ray Detector Software (XDS) using XDSapp^42,43^. The Sirt6 crystals were detected to be twinned through L-tests in POINTLESS^44^. Structures were solved by molecular replacement phasing with Phaser^45^ from the CCP4 software suite^46^, using the twinned data and a Sirt6/ADP-ribose structure (PDB code 3K35)^31^ as a search model for the Sirt6 complexes and Sirt2/1,2,4-Oxadiazole/ADP-ribose structure (PDB code 5MAR)^47^ for the Sirt2 complex. The structures were manually rebuilt in COOT^48^ and refined with Refmac^49^. The refinements were done with the amplitude-based twin refinement option and yielded twin fractions of 22–44% (Table 1). Structure figures were generated with PyMOL (Schrödinger, LLC; https://pymol.org/2/).

## Supplementary information

Supplementary information

## Data Availability

**Co-ordinates**. X-ray diffraction data and coordinates have been deposited with the wwPDB (www.wwpdb.org) under accession numbers 6QCD (Sirt6/quercetin), 6QCN (Sirt2/quercetin), 6QCJ (Sirt6/catechin gallate), 6QCH (Sirt6/cyanidin), 6QCE (Sirt6/isoquercetin).
