# Supplementary material for: Structural basis for the activation and inhibition of Sirtuin 6 by quercetin and its derivatives
Source: Sci Rep. 2019 Dec 16;9:19176. doi: 10.1038/s41598-019-55654-1 (PMC6914789; doi:10.1038/s41598-019-55654-1)
Supplement: Supplementary file 1 — Supplementary information [file 41598_2019_55654_MOESM1_ESM.pdf]

Supplementary Information

Structural basis for the activation and inhibition of Sirtuin 6 by quercetin and its derivatives

Weijie You[1], Wei Zheng[2,3], Sandra Weiss[1], Katrin F. Chua[2,3], Clemens Steegborn[1]

[1]Department of Biochemistry, University of Bayreuth, 95445 Bayreuth, Germany

[2]Department of Medicine, Stanford University School of Medicine, Stanford, CA 94305, USA

[3]Geriatric Research, Education, and Clinical Center, Veterans Affairs Palo Alto Health Care System, Palo Alto, CA 94304, USA

Supplementary Figure 1

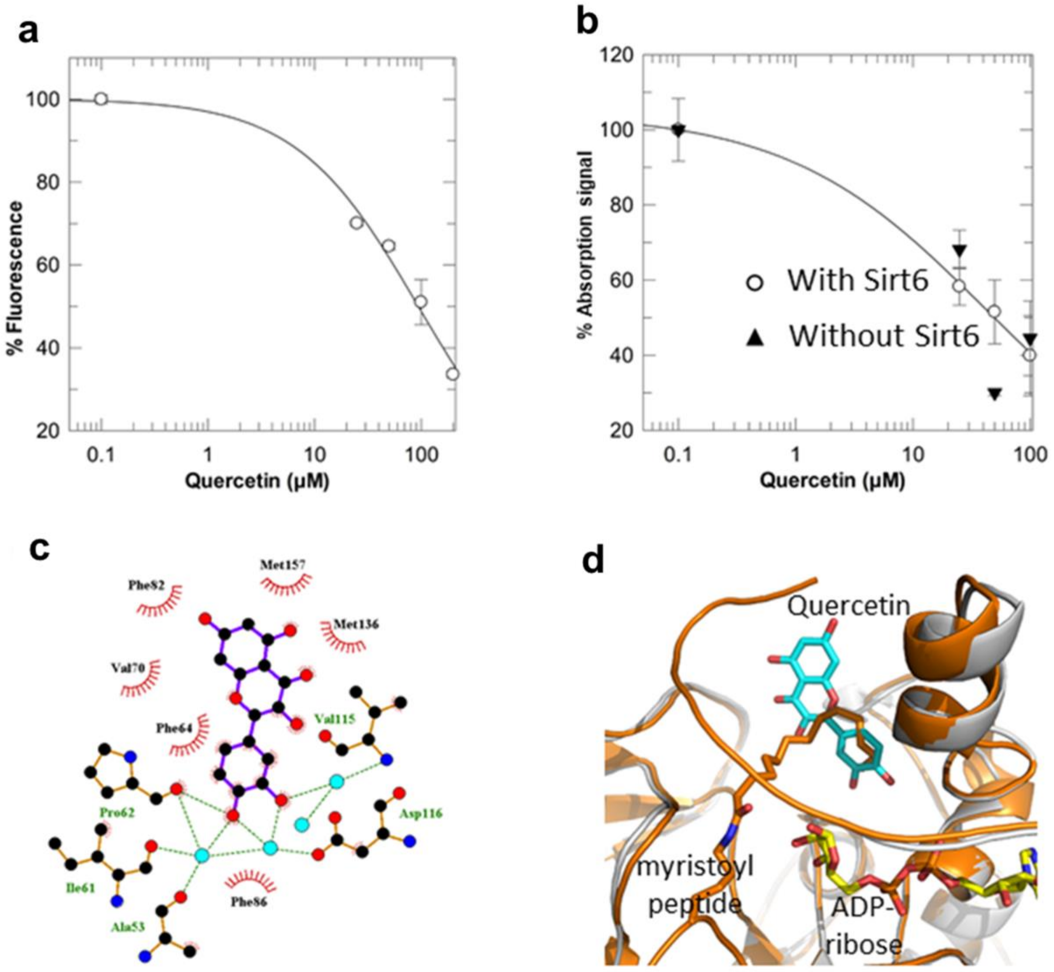

Supplementary Figure 1: (a) Dose-dependent effects of quercetin on the fluorescence signal in control reactions for the FdL assay. (n = 3; error bars: s.d.) **(b)** Quercetin titration yields concentration dependent inhibitory effects in coupled enzymatic assays in Sirt6 samples (○) but also in control reactions without Sirtuin (▲). (n = 3; error bars: s.d.) **(c)** Schematic view of the interactions between Sirt6 and quercetin. **(d)** Overlay of the Sirt6/quercetin complex (gray cartoon, cyan ligand) with a Sirt6/myristoyl-peptide complex (orange cartoon and peptide, PDB code 3ZG6).

Supplementary Figure 2

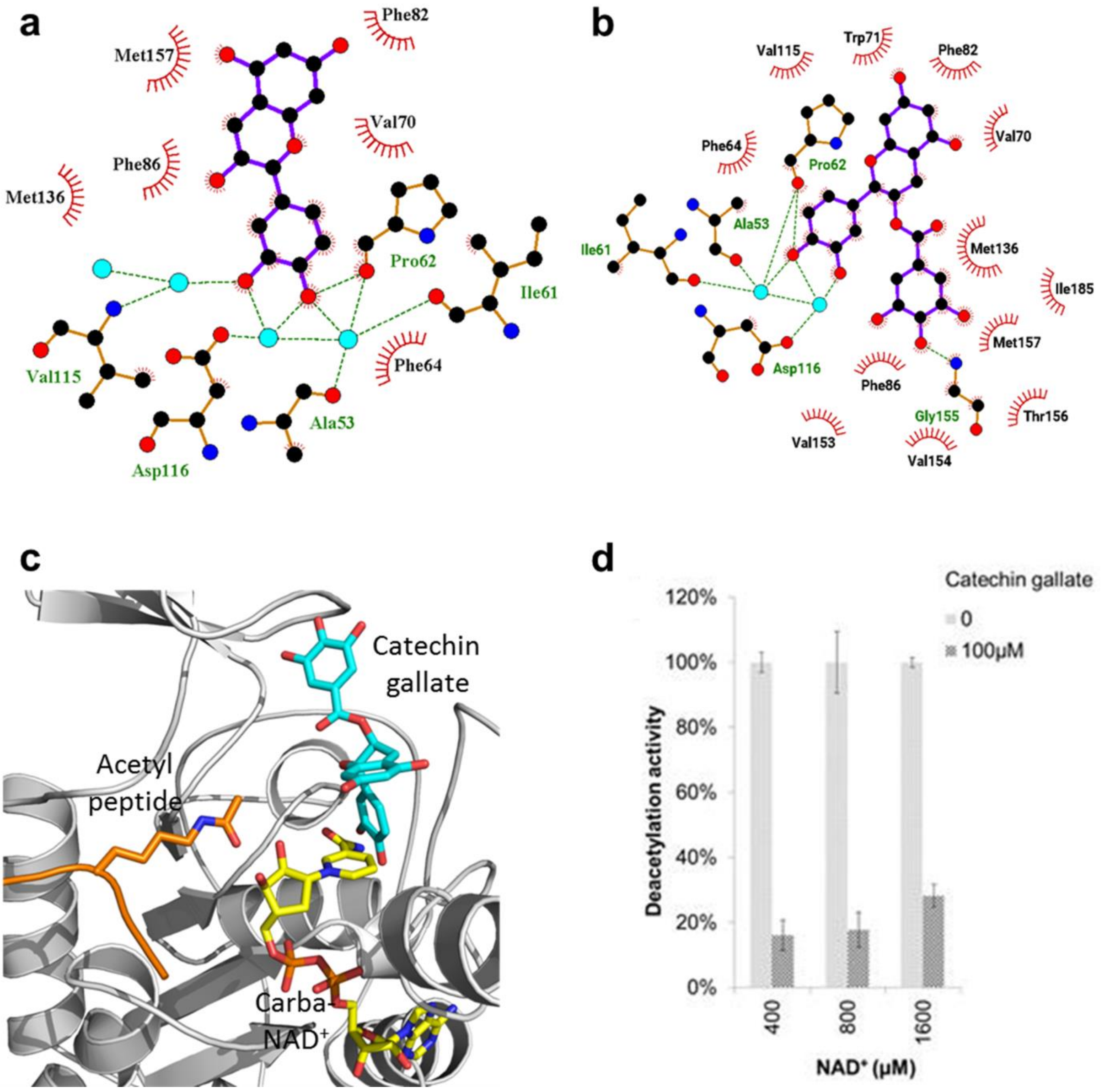

Supplementary Figure 2: (a) Schematic view of the Sirt6/cyanidin interactions. **(b)** Schematic view of the interactions between Sirt6 and CG. **(c)** Overlay of the Sirt6/ADP-ribose/CG complex (gray cartoon, cyan ligand) with a Sirt3/acetyl-ACS peptide/carba-NAD^+ complex (PDB ID 4FVT; protein hidden, peptide displayed in orange and carba-NAD^+ as yellow stick). **(d)** Inhibition of Sirt6 deacetylation activity by 100 µM CG at different NAD^+ concentrations. (n = 3; error bars: s.d.)

Supplementary Figure 3

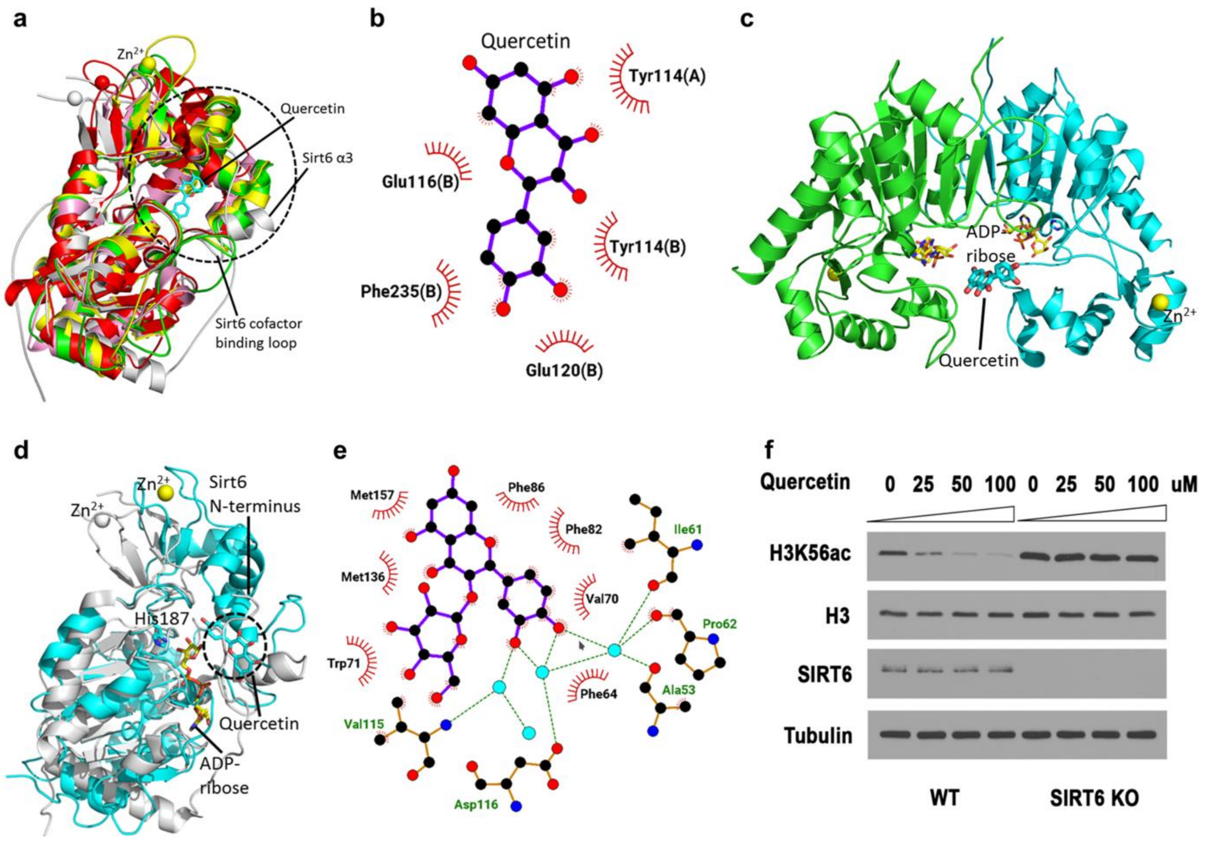

Supplementary Figure 3: (a) Overlay of Sirt6/ADP-ribose/quercetin (gray protein, quercetin as cyan stick) with Sirt1 (yellow; PDB ID 4IF6), Sirt2 (green; PDB ID 3ZGV), Sirt3 (pink; PDB ID 4FVT) and Sirt5 (red; PDB ID 4G1C). Dotted circle: Cofactor-binding loop and neighboring helix bundle. **(b)** Schematic view of the Sirt2/quercetin interactions. **(c)** Overall structure of the human Sirt2/ADP-ribose/quercetin complex (green/cyan cartoon of two symmetry-related monomers). ADP-ribose (yellow) and quercetin (cyan) are shown as sticks. **(d)** Overlay of the Sirt2/quercetin complex (cyan/green) with the Sirt6/quercetin complex (gray). Dotted circle: steric clash between Sirt6 N-terminus and quercetin. **(e)** Schematic view of the Sirt6/isoquercetin interactions. **(f)** Histone deacetylation activity in cells. After treatment with quercetin at indicated doses for 48 hours, acetylation levels of H3K56 were analysed by Western blots. Blots were cropped for detection of the indicated entities.

Supplementary Figure 4

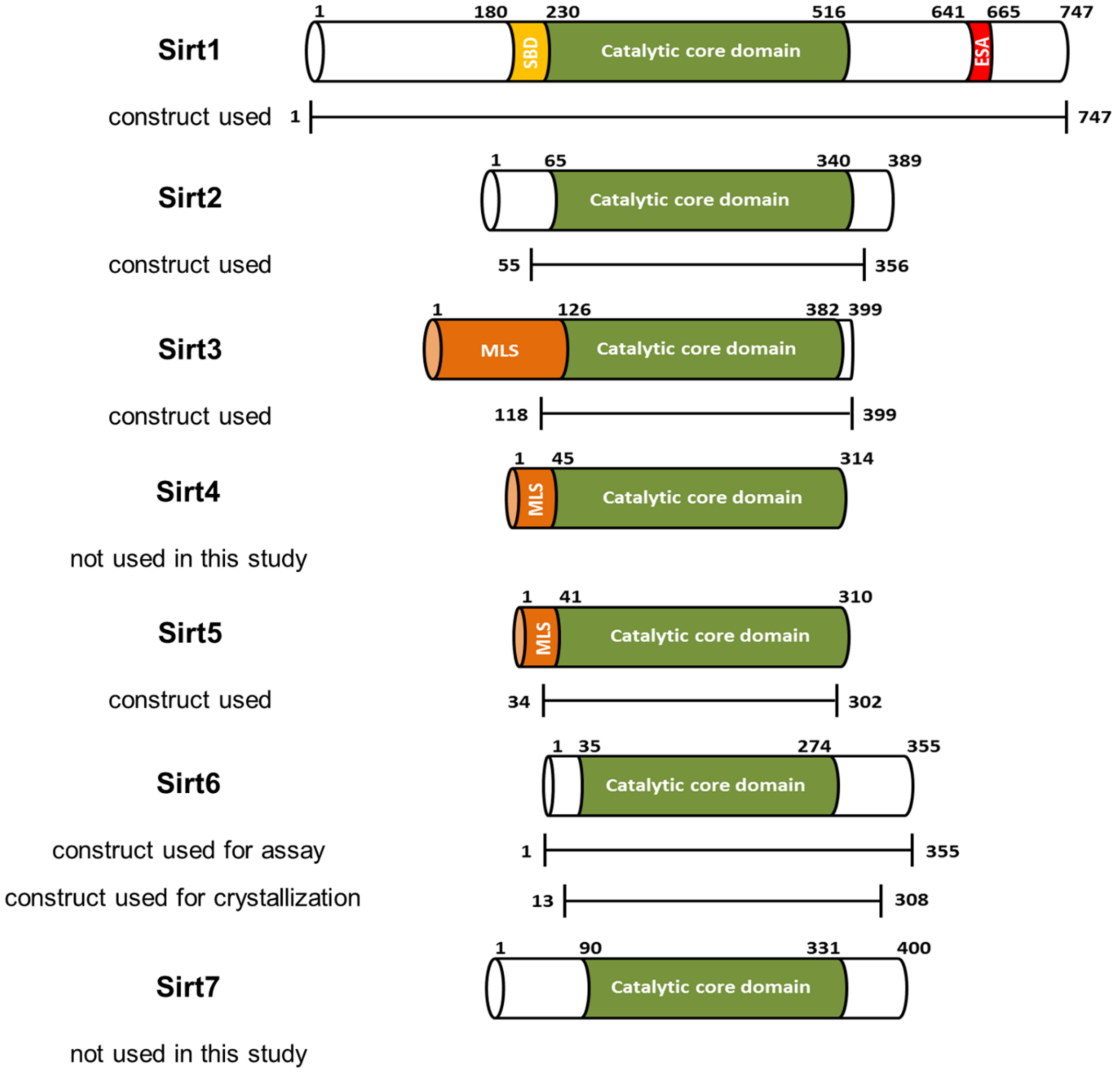

Supplementary Figure 4: Schematic view of the architecture of human Sirtuin isoforms and the protein constructs used in this study. The conserved Sirtuin catalytic core is shown in green, the Sirt1-specific “Sirtuin Activating Compound (STAC) Binding Domain” (SBD) in yellow. The “Essential for Sirt1 Activity” ESA domain is shown in red, and the “Mitochondrial Localization Sequence” (MLS) of Sirt3, 4, and 5 in orange. The protein constructs used in this study are indicated below each isoform.
